# Long-Term In Vivo Administration of Panaxynol Alleviates Diabetes-Induced Vascular Calcification by Modulating Sirt6-Mediated sEH Function in Perivascular Adipose Tissue

**DOI:** 10.1021/acs.jafc.5c01982

**Published:** 2025-07-07

**Authors:** Shanshan Song, Xina Yu, Changming Xie, Zhanhua Li, Ying Zhang, Yan Liu, Zhongjue Qiu, Tiantian Wang, Hongna Su, Hui Huang, Pei Luo

**Affiliations:** ‡ State Key Laboratory for Quality Research in Chinese Medicines, Joint Laboratory of Guangdong−Hong Kong−Macao Universities for Internationalization of TCM, Macau University of Science and Technology, Macau 999078, China; § Department of Cardiology, The Eighth Affiliated Hospital, Joint Laboratory of Guangdong−Hong Kong−Macao Universities for Nutritional Metabolism and Precise Prevention and Control of Major Chronic Diseases, Sun Yat-sen University, Shenzhen 518033, China; ⊥ Department of Anesthesiology, The Affiliated TCM Hospital of Southwest Medical University, Luzhou 646000, China; ∥ Guangxi Liuyao Group Company, Ltd., Liuzhou 545001, China; ¶ School of Biological and Chemical Engineering, NingboTech University, Ningbo 315100, China

**Keywords:** panaxynol, diabetes mellitus, perivascular adipose tissue, soluble epoxide hydrolase, Sirt6

## Abstract

Panaxynol (PA), a bioactive compound found in carrots and other Apiaceae plants, holds promise for managing diabetes-related vascular complications. This study investigated the long-term effects of PA on diabetes-induced vascular calcification (DVC) in db/db mice for up to 9 weeks. The impact of PA on DVC was evaluated both in vivo and in vitro using immunohistochemical staining, Western blotting, micro-computed tomography, and other analytical techniques. Results demonstrated that PA significantly attenuated arterial calcification and collagen hyperplasia in the aorta. Additionally, PA elevated levels of the lipid signaling molecule 14,15-EET, which preserved the vascular structure and function by reducing inflammatory macrophage infiltration in perivascular adipose tissue. This protective effect was partially attributed to the PA-mediated inhibition of soluble epoxide hydrolase (sEH) through Sirt6 activation. These findings highlight PA as a potential natural therapeutic strategy for mitigating diabetic vascular complications and offer an alternative to conventional pharmacological approaches.

## Graphical abstract

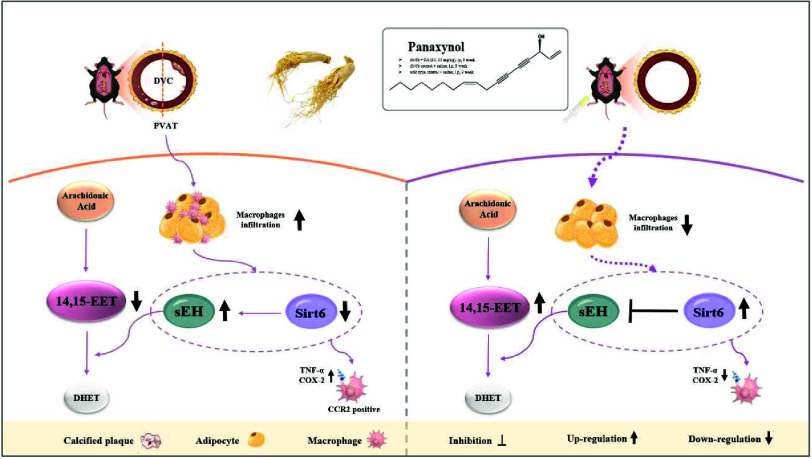

## 1 Introduction

Vascular calcification (VC), characterized by the accumulation of hydroxyapatite mineral deposits on existing fatty plaques within blood vessels, contributes to vascular narrowing and poses a significant threat to individuals with diabetes mellitus (DM). Clinical observations have consistently revealed a strong association between DM and VC, as evidenced by a greater prevalence of VC in diabetic patients than in those without diabetes.^1,2^ While VC can manifest in various vascular beds, it is particularly concerning when it affects the aorta, where it is most commonly observed and acts as a strong independent predictor of cardiovascular events. However, current antidiabetic therapies (including metformin, sulfonylureas, thiazolidinediones, sodium–glucose cotransporter protein-2 inhibitors, etc.) demonstrate limited efficacy against diabetes-induced vascular calcification (DVC), while having a greater risks of heart failure, lactic acidosis, genital infection, or amputation.^3–8^ Therefore, considering the significance and burden of VC, it is very important to consider diet supplements to minimize the development of DVC in clinical treatment.

Panaxynol (PA), a fat-soluble bioactive ingredient of ginseng that also occurs in carrot and celery, belongs to the polyacetylene compound class.^9^ PA exhibits a variety of biological activities, including the inhibition of vascular smooth muscle cell proliferation and migration,^10^ antiplatelet agglutination,^11^ and antihypertensive effects,^12^ suggesting its potential for preventing VC. Multiple pharmacological and pharmacokinetic studies have further demonstrated the lack of toxic side effects of PA in mice, underscoring its favorable safety characteristics for use and provided enough margin of doses for the long-term intervention.^13–16^ Metabolic disorders in DM lead to inflammation and oxidative stress, resulting in endothelial dysfunction and arterial injury. PA has been previously shown to have antioxidant and antiinflammatory properties,^17,18^ making it a promising candidate for vascular protection in diabetes-related conditions. However, the specific function of PA in mitigating VC in the context of DM and the underlying mechanisms are still unknown.

Chronic hyperglycemia-induced reactive oxidative stress and inflammatory responses play pivotal roles in the development of VC in DM.^19,20^ Perivascular adipose tissue (PVAT), considered a secretory organ, exerts a regulatory influence on the vasculature. In metabolic disease states, PVAT-derived cytokines or inflammatory factors can interact with the artery wall to regulate vascular tone, inflammation, and vascular remodeling via paracrine action.^21–23^ Cytochrome P450 (CYP) enzyme-derived epoxyeicosatrienoic acids (EETs) serve as crucial lipid signaling molecules involved in the regulation of angiotasis, inflammation, and tissue regeneration, and their activity is subject to modulation by soluble epoxide hydrolase (sEH), which converts EETs into less active dihydroxyeicosatrienoic acids (DHETs).^24,25^ Hence, stabilizing endogenous EETs via sEH inhibition represents a potential strategy for regulating various vascular pathophysiological processes. Clinical studies have linked a decrease in (±)­14(15)-epoxy-5*Z*,8*Z*,11*Z*-eicosatrienoic acid (14,15-EET) in patients to an increased incidence of VC,^26^ suggesting that 14,15-EET is a potential target for VC intervention, albeit through an unclear mechanism. In general, DM is characterized by insulin secretion and action defects, accompanied by the recruitment of inflammatory cells, contributing to lipid metabolic abnormalities, which underlie atherosclerosis. Although the association between VC and PVAT has been established,^27,28^ the function of PVAT-derived sEH in the development of DVC remains to be elucidated. This study aimed to explore the potential therapeutic effects of PA in managing DVC and to elucidate the underlying mechanism, focusing on adipocyte-derived sEH regulation in db and db mice and 3T3-L1 adipocytes.

## 2 Materials and Methods

### 2.1 Materials and Reagent

Dulbecco’s modified Eagle medium (DMEM)/F12, penicillin–streptomycin–glutamine (PSG), and fetal bovine serum (FBS) (Gibco, North Andover, MA); 3-isobutyl-1-methylxanthine (IBMX), insulin, indomethacin, rosiglitazone, and dexamethasone (Macklin, Shanghai, China); thiazolyl blue tetrazolium bromide (MTT) (BBI Solutions, Wales, U.K.); dimethyl sulfoxide (DMSO), acetonitrile, methyl *tert*-butyl ether (MTBE) (Aladdin, Shanghai, China); bovine serum albumin (BSA; Sigma-Aldrich, St. Louis, MO); sEH assay kit (Abcam, Cambridge, U.K.); RIPA lysis buffer (Beyotime, Shanghai, China); phosphatase inhibitor (Thermo, Waltham, MA); sodium dodecyl sulfate polyacrylamide gel electrophoresis (SDS-PAGE) loading buffer (CWBIO, Jiangsu, China); poly­(vinylidene difluoride) membranes (Millipore Corp., Billerica, MA); protease–phosphatase inhibitors (Thermo, Waltham, MA); antibodies Sirt6, F4/80, and CD11b (Abcam, Cambridge, U.K.), CCR2 (Novus Inc., Tarzana, CA), sEH and COX-2 (Santa Cruz, CA), α-tubulin and β-actin (CST, Danvers, MA); UltraSignal ECL Chemiluminescent Substrate (4A biotech, Beijing, China); Oil Red O (Sigma-Aldrich, St. Louis, MO); ethanol and isopropyl alcohol (Sinopharm Chemical Reagent Co., Ltd., Shanghai, China); 4% paraformaldehyde (PFA; Biosharp, Beijing, China); adipose-tissue fix-solution (Servicebio, Hubei, China); trinitrophenol (Sigma-Aldrich, St. Louis, MO); Verhoeff–van Gieson stain kit (HaoKe Biotechnology, Zhejiang, China); Picrosirius Red (Solarbio, Beijing, China); (±)­14(15)-epoxy-5*Z*,8*Z*,11*Z*-eicosatrienoic acid (14,15-EET; Cayman Chemical, Ann Arbor, MI); cDNA synthesis Super Mix, SYBR Green qPCR superMix kit (Transgen, Beijing, China).

### 2.2 Cytotoxicity, Adipocyte Differentiation, and Lipid Content Assays In Vitro

To generate adipocytes,^29^ 3T3-L1 cells (FH0359, Biological Technology Co., Ltd., Yantai, China) were seeded in 24-well plates (2 × 10^5^ cells/well) and incubated at 37 °C for 24 h to enable the cells to adhere and multiply until they stopped growing due to contact inhibition for 48 h in DMEM/F12 containing 10% FBS and 1% PSG. Then, the cells were induced by a differentiation cocktail, which included 0.5 mM IBMX, 0.2 mM indomethacin, 1 μM dexamethasone, 10 μg/mL insulin, and 1 μM rosiglitazone, within the medium in an incubator containing 5% CO_2_ at 37 °C for 2 or 3 days. Then, the medium was replaced with DMEM/12 containing 10% FBS, 1% PSG, 10 μg/mL insulin, and 1 μM rosiglitazone for another 3–6 days. The cell differentiation efficiency was validated by Oil Red O staining. The intracellular TG content in the samples was assessed with a GPO-PAP ELISA kit after disrupting the cells using an ultrasonic cell crushing machine on ice.^30^

The cytotoxic effect of PA was detected by MTT assay, and mature 3T3-L1 adipocytes were induced in three 24-well plates as previously described.^31^ Next, the plates were washed with PBS and treated with gradient concentrations of PA (1–100 μM; the ^1^H and ^13^C NMR spectroscopic data of PA are shown in Figure S1 and Table S1) in full DMEM/F12 (the medium was replaced every 2 days). The medium was replaced with a fresh medium supplemented with 100 μL of MTT solution (5 mg/mL) at 2, 4, and 7 days, and the plates were incubated for another 4 h in the dark. The formazan crystals of the mitochondrial matrix were dissolved in DMSO after the supernatant. The optical density was determined spectrophotometrically at 570 nm by using a microplate reader (Molecular Devices, San Jose, CA).

### 2.3 sEH Activity Assay

Intracellular sEH enzymatic activity was determined using an sEH assay kit. The fluorescent standard sample was prepared in accordance with the instructions. The adipocyte samples were thoroughly mixed with 100 μL of chilled sEH assay buffer and lysed on ice for 10 min, followed by centrifugation at a force of 10000*g* for 15 min at 4 °C. The supernatant was collected, and then the protein concentrations were estimated using a BCA protein quantitation kit. On the other hand, lysates were precipitated by adding 80% saturated (NH_4_)_2_SO_4_ for 30 min. For activity analysis, the pellet that had been centrifuged and cleaned was again suspended in an equivalent volume of assay buffer. Samples with or without an inhibitor were added to each well of a clear 96-well plate and incubated for 10 min. Then, assay buffer or reaction mix buffer (containing an sEH substrate) was added to the wells at the same time. Immediately, the fluorescence (Ex/Em 362/460 nm) was recorded in kinetic mode for 20 min, and the standard curve was read in the end-point mode. sEH activity was calculated using the following kit instructions.

### 2.4 Animals and PA Treatment

Male db/db mice (Lepr^db^; BKS. Cg-Dock 7 m + /+Lepr^db^ J, JAX 000642, black and obese, 7 weeks old) and their wild-type (WT) control littermates were purchased from Gempharmatech Co., Ltd. (Jiangsu, China). All mice were kept in a clean environment with a standard diet and housed in a consistent setting (12 h light/dark cycle, 40–50% humidity at 25 °C). Following a 2-week adaptation period, 9-week-old mice were randomly allocated to four groups: two db/db groups received PA treatment (30 or 15 mg/kg, i.p.) every 3 days over a 9-week span, while the db/db control and WT control plants were treated with saline for comparison. PA was suspended in PEG400 with a brief vortexing for 30 s, followed by the addition of physiological saline. The PA solution was prepared freshly on ice prior to each treatment session. Before sacrifice by diethyl ether asphyxiation, fasting blood glucose levels were detected via tail-tip blood with a hand-held glucometer (ACCU-CHEK, Roche Diagnostics Corp., Shanghai, China) at the end of week 18. Whole blood, PVAT, aorta, heart, liver, spleen and kidney were collected and stored at −80 °C or immediately fixed in 4% PFA. The animal experiments were approved by the Department of Health and Supervision of the Municipal Affairs Bureau and the Animal Ethics Committee of Macau University of Science and Technology (No. AL001/DICV/DIS/2021). All experiments in this study were conducted according to the animal protection legislation of the Macau Special Administrative Region (4/2016) and the ARRIVE guidelines of the Laboratory Animal Center at Macau University of Science and Technology.

### 2.5 Calcification and Lesion Analysis

The aortic tissues were fixed in 4% PFA. After 48 h, the aortas were exposed for digital imaging. The calcium content in the entire aorta was quantified using a modified method based on digital X-ray analysis on a micro-computed tomography (micro-CT) scanner (SkyScan 1176, Bruker, Billerica, MA). The parameters used were as follows: 248 μA current, 40 kV voltage, 12.28 μm camera pixel size, 16.88 μm image pixel size, 0.500° rotation step, and 185 ms exposure without a filter. While undergoing vertical axis rotation, the aorta was subjected to three-dimensional reconstruction using CTvox (version 3.0) and Nrecon (version 1.6.10.4) for further analysis. Finally, two-dimensional thresholding was applied to the segmentation image, and the calcifications were detected and marked in the aorta using CTAn (version 1.15). The aortic calcification score (AoCS) was quantified by calculating the ratio of the calcified volume (mm^3^) to the aorta volume (mm^3^), which was then multiplied by 100 and reported in arbitrary units.^32^

### 2.6 Oil Red O, Picrosirius Red, and Verhoeff–van Gieson Staining of the Aorta

The lesions in the en face aortas were determined using Oil Red O staining.^33^ Picrosirius Red and Verhoeff–van Gieson stains were applied to 5-μm cross sections of the aortic arch to determine the collagen content and thickness of the intima–media layers as previously described.^34,35^ After staining, all samples (en face aortas or the cross sections of the aortic arch) were taken with an inverted biological microscope (BX63, Olympus, Tokyo, Japan). The images were quantified by two individuals who were blinded to the experimental design and each other’s result using a computer-assisted image analysis protocol. The en face lesion areas were expressed as a mean percentage of the Oil Red O^+^ area in the aorta plus.

### 2.7 Western Blot

Mouse PVAT and differentiated 3T3-L1 adipocytes were homogenized on ice in RIPA lysis buffer (containing protease–phosphatase inhibitors) to prepare the lysates. The supernatants were obtained by centrifuging the lysates at 14000*g* for 10 min at 4 °C, after which the total protein concentration was quantified with a BCA assay kit. The equivalent amounts of denatured lysate proteins from each biological replicate were separated via 8–10% SDS-PAGE and transferred to poly­(vinylidene difluoride) membranes. The membranes were then blocked in 5% BSA in Tris-buffered saline with Tween-20, incubated with primary antibodies overnight at 4 °C, and then incubated with horseradish peroxidase (HRP)-conjugated secondary antibodies for 1 h at room temperature in a blocking solution. An Amersham Imager system was used to detect the protein bands with an UltraSignal ECL chemiluminescent substrate.

### 2.8 Immunohistochemistry (IHC)

IHC was used to assess adipose tissue macrophage populations according to previous reports.^36^ The fixed samples were paraffin-embedded, and sequential micrometer sections were mounted on slides. After being dewaxed, antigen repaired, and hydrogen peroxidase blocked, the slides were incubated with primary antibodies against CD11b and F4/80 overnight at 4 °C to assess macrophages. After 15 min of incubation in 0.3% H_2_O_2_, HRP-conjugated secondary antibodies were added to the slides, which were allowed to react at room temperature for 1 h. Then, 3,3′-diaminobenzidine chromogen (1:19) and hematoxylin (1:1) counterstain were used for detection. After IHC processing, the slides were rinsed, dehydrated, cleared, and mounted. The slides were visualized by using an inverted biological microscope. The samples in each group were randomly captured by two independent observers. The integrated optical density (IOD) values were quantified using Image-Pro plus (version 6.0), and the final results were expressed as the mean optical density (MOD, where MOD = IOD/total tissue area) of positively stained macrophages in two different views.

### 2.9 Gene Expression Analysis

TRlzol reagent was utilized to extract total RNA from adipocytes or adipose tissues, followed by the synthesis of cDNA using an all-in-one first-strand cDNA synthesis Super Mix. qPCR analysis was conducted via a StepOnePlus real-time PCR machine (ViiA 7) from Thermo Fisher Scientific using a SYBR Green kit according to the manufacturer’s directions, and the relative expression of all genes was normalized to that of β-actin via the 2^–ΔΔCt^ method. All PCR primer sequences are listed in Table S3.

### 2.10 Sample Preparation, Instrumentation, and UHPLC-QqQ-MS/MS Conditions

Arachidonic acid metabolites from blood serum were obtained through the liquid–liquid extraction method as follows: a mixture of serum (200 μL) and MTBE (800 μL) in a 1.5 mL microtube was vortexed for 5 min and centrifuged at 10000 rpm for 5 min (4 °C). Then, the sediments were obtained from the dried supernatant (under a nitrogen stream at 4 °C). Before UHPLC-QqQ-MS/MS analysis, the sediments were reconstituted in 50 μL of an acetonitrile/water/acetic (50:50:0.02, v/v/v) solution. A predetermined standard of the reference compound (14,15-EET) was dissolved in methanol to obtain a stock solution of 14,15-EET (1 μg/mL), which was subsequently diluted to obtain working standard solutions (50–0.0625 ng/mL). 14,15-EET was quantified by an Ultra-HPLC system with a 0.2 mL/min flow rate and a 2 μL sample volume for 10 min using an ACQUITY HSS T3 column at 35 ± 2 °C and eluted with a gradient concentration of water (0.03% Hac) (A) and acetonitrile (B) as previously reported.^37^ The electrospray ionization mass spectrometry system was operated under the following conditions: gas temperature, 350 °C; gas flow, 10 L/min; sheath gas, 350 °C, 11 L/min; pressure, 35 psi; ion spray, −3500 V.

### 2.11 Statistical Analysis

Biological characteristics were evaluated via one-way ANOVA or two-way ANOVA tests unless they were not normally distributed, in which case independent-sample Kruskal–Wallis tests were used. Every trial was carried out in triplicate, and the results are shown as the mean ± SEM; *p* < 0.05 was considered to indicate statistical significance.

## 3 Results

### 3.1 Inhibition of sEH Activity by PA

Previous studies have highlighted sEH as a promising target for treating diabetes, inflammation, and hypertension.^38^ To assess the effect of PA on sEH within adipocytes, we utilized the 3T3-L1 cell line to establish a mature adipocyte model. We observed a substantial increase in the number of lipid droplets in differentiated 3T3-L1 adipocytes before PA treatment (Figure 1A). Subsequently, adipocytes were treated with different concentrations of PA to evaluate its cytotoxicity and impact on the lipid content. As depicted in Figure 1B, PA exhibited a concentration- and time-dependent reduction in cell viability, with a significant decrease observed at 50 and 100 μM PA (*p* < 0.01 and 0.001) on day 2. This cytotoxicity persisted with prolonged exposure, especially at concentrations exceeding 12.5 μM. However, within a noncytotoxic dose range, PA significantly reduced sEH mRNA levels, nearly halving its expression at the transcriptional level in the 1 and 6.25 μM groups (*p* < 0.05) (Figure 1C). Conversely, changes in the sEH protein levels were negligible (Figure S3). Furthermore, PA had an inhibitory effect on sEH activity within the cells, with the 2 μM group showing significant inhibition (*P* < 0.05) (Figure 1D). These findings indicate that PA inhibits sEH in differentiated 3T3-L1 adipocytes.

**Figure 1:**
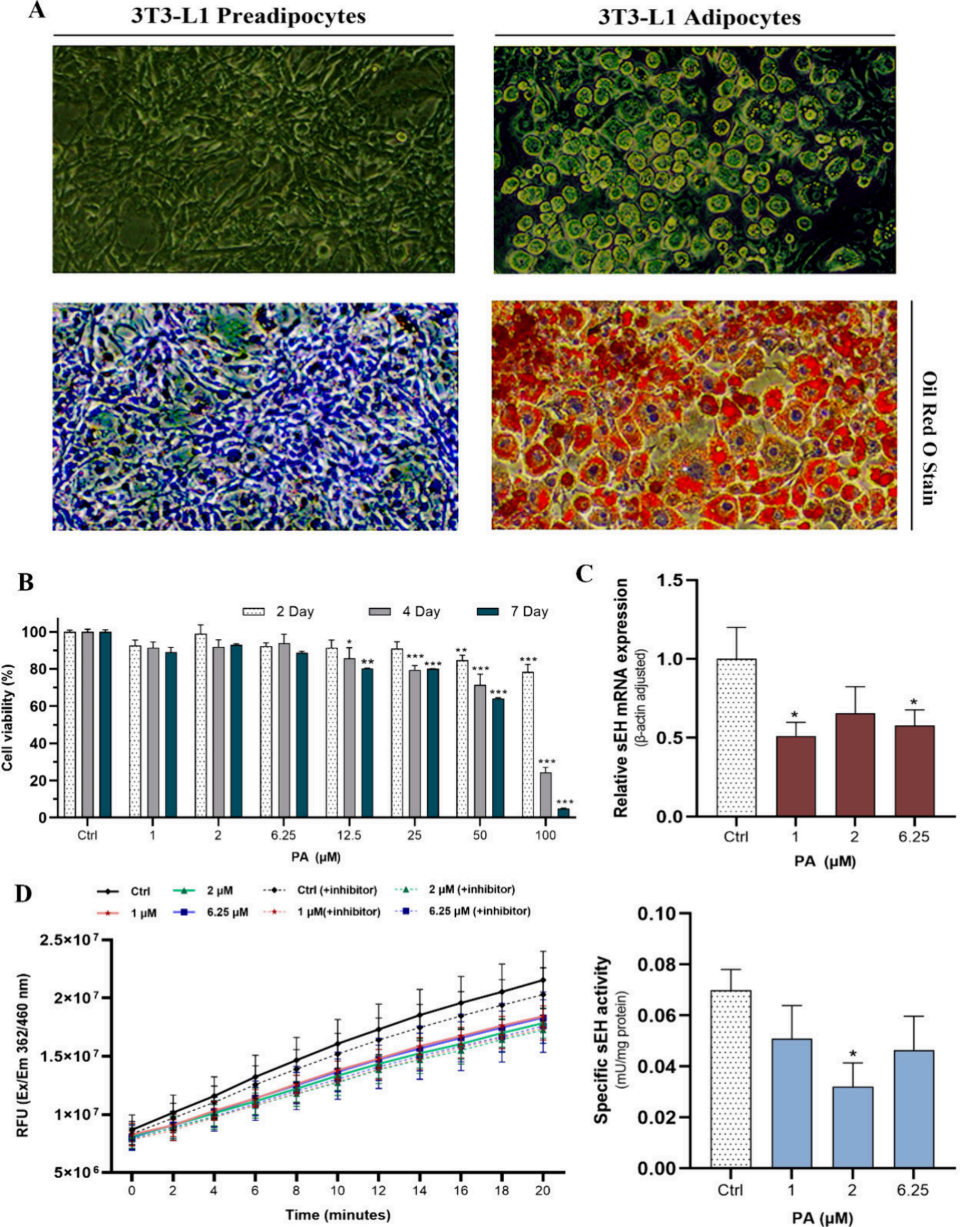
Effect of PA on 3T3-L1 adipocytes. (A) Representative illustration of adipocyte differentiation. (B) Assessment of the cytotoxicity of PA on mature 3T3-L1 adipocytes. (C) Quantification of relative mRNA levels of sEH. (D) Enzymatic activity of sEH. Two-way ANOVA (cell viability) with Tukey’s multiple comparisons test and one-way ANOVA (specific sEH activity) with uncorrected Fisher’s LSD test were performed for the data that followed the normal distribution. The Kruskal–Wallis test was performed with uncorrected Dunn’s test for data that did not follow the normal distribution (sEH mRNA). All data are represented by statistical significance of mean ± SEM (*n* = 3 for all groups). *p < 0.05, **p < 0.01, ***p < 0.001, and *p* > 0.05 n.s.

### 3.2 PA-Mediated Enhancement of Serum 14,15-EET via sEH Inhibition in db/db Mice

To investigate the effect of PA on sEH in vivo, we utilized db/db mice as a model and administered PA, as illustrated in Figure 2A. Comparing PVAT from db/db mice to that from WT control mice revealed a significant increase in sEH expression. However, PA treatment, particularly at 30 mg/kg (*p* < 0.01) for transcriptional levels and 15 mg/kg (*p* < 0.01) for protein levels, reduced sEH expression (Figure 2B,C). These observations suggest that PA has an inhibitory effect on sEH. Subsequently, serum 14,15-EET levels were detected by UHPLC-QqQ-MS/MS (Figure 2D). We observed a significant reduction in the 14,15-EET levels in db/db mice (1.657 ± 1.060 ng/mL) compared to those in WT control mice (5.445 ± 3.421 ng/mL) (*p* < 0.01). However, PA treatment led to an increase in the 14,15-EET levels in the db/db groups, reaching 3.439 ± 1.587 and 2.677 ± 1.877 ng/mL in the 30 and 15 mg/kg groups, respectively. The above results further support the opinion that PA inhibits PVAT-derived sEH activity, leading to increased 14,15-EET levels in db/db mice.

**Figure 2:**
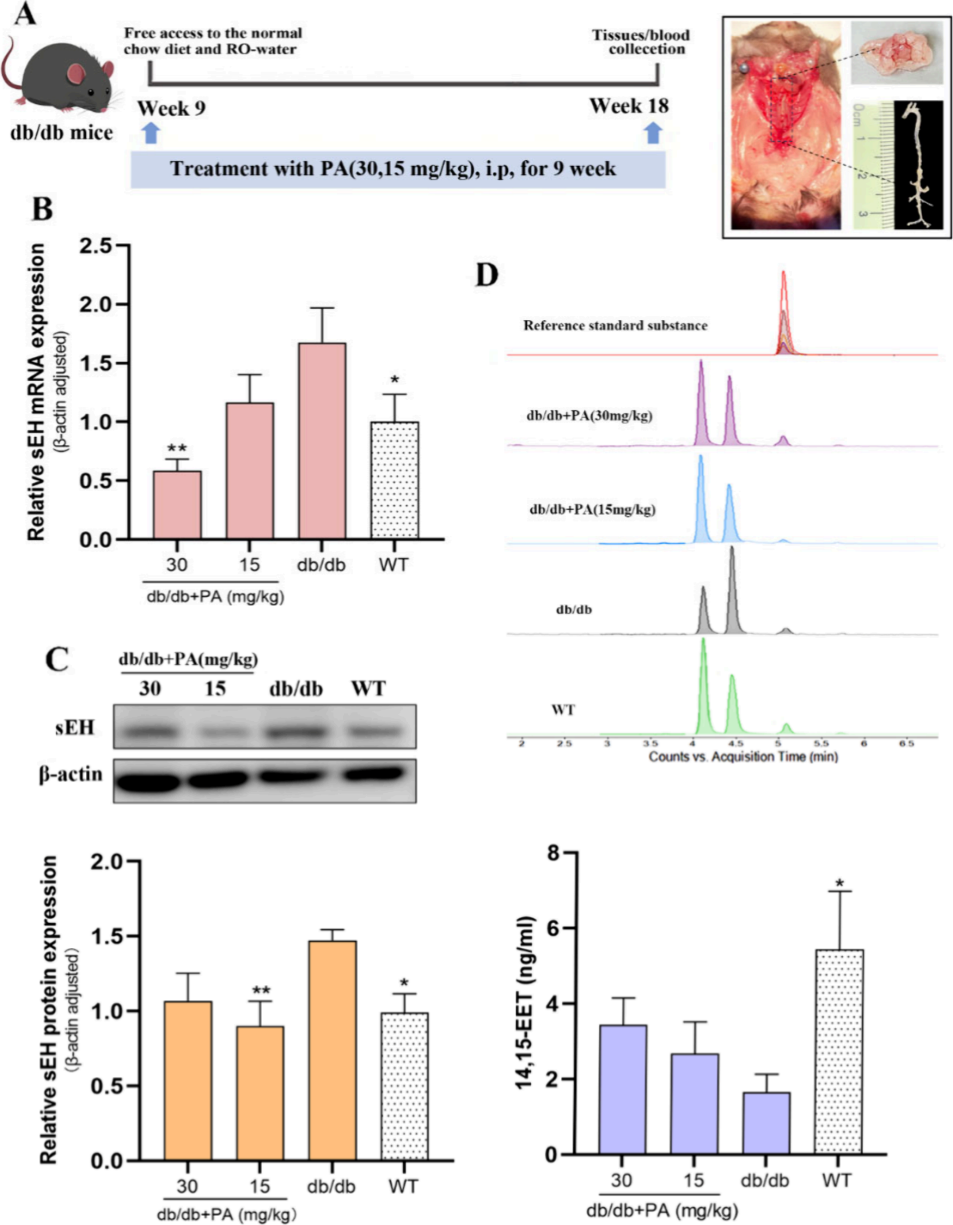
Characterization of sEH expression in PVAT and serum 14,15-EET content in db/db mice. (A) Experimental treatment overview. (B) mRNA expression of sEH (*n* = 6). (C) Protein expression of sEH (*n* = 7). (D) Measurement of the 14,15-EET levels (*n* = 5). One-way ANOVA with uncorrected Fisher’s LSD test (sEH protein, 14,15-EET) was performed for the data that followed the normal distribution. The Kruskal–Wallis test was performed with uncorrected Dunn’s test for data that did not follow the normal distribution (sEH mRNA). All data are represented by statistical significance of mean ± SEM. *p < 0.05, **p < 0.01, ***p < 0.001, #*p* < 0.05, ##*p* < 0.01, ###*p* < 0.001 vs WT, and *p* > 0.05 n.s.

### 3.3 PA Treatment Ameliorates VC Changes in db/db Mice

We also investigated the influence of PA on aortic morphological and pathological indices in db/db mice. Micro-CT analysis, with a resolution of 12.28 μm, revealed predominant calcification in the aortic arch, with minimal deposits in the ventral aorta or descending aorta (Figure 3A). The AoCS of the db/db mice significantly exceeded that of the WT mice. Surprisingly, the calcification levels markedly decreased after PA treatment. Compared to those of untreated db/db mice, the mean calcification scores were reduced from 0.0553 to 0.0238 and 0.0122 in the 30 and 15 mg/kg groups, respectively (Figure 3B). Oil Red O staining demonstrated that lipid deposition mainly occurred in the aortic arch of db/db mice, consistent with the CT results. PA treatment led to a substantial decrease in the number of Oil Red O-positive areas in the aorta, which approached that in the WT group (Figure 3C,D). Additionally, collagen hyperplasia and disruption of elastic fibers were observed in db/db mice, along with a thickening of the intima–media layers. Moreover, PA treatment partially restored the wavy structure of the elastic lamellae and decreased the collagen content (Figure 3E,H). While db/db mice exhibited significant weight gain, organ hypertrophy, and hyperglycemia, PA intervention led to slowed weight gain and ameliorated fasting blood glucose levels, indicating potential benefits in mitigating hyperglycemia (Figure 3I,J). However, no significant improvements were observed in the heart, liver, or spleen mass (relative to body weight) ratios under PA treatment (Figure S5). Together, these results demonstrate that PA attenuates arterial calcification and associated pathological changes linked to diabetes development.

**Figure 3:**
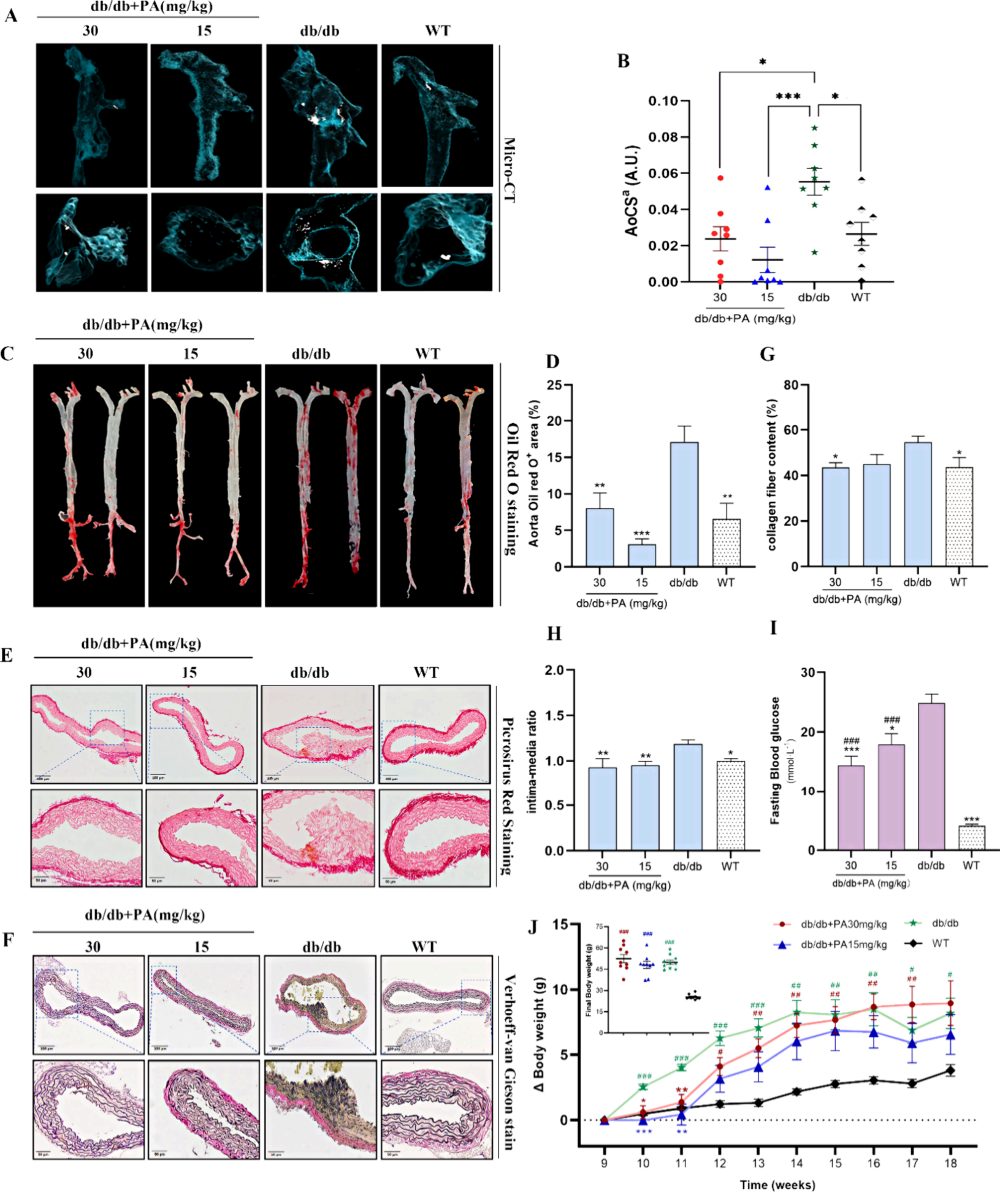
Effect of PA on aortic morphological and physiological indexes in db/db mice. (A and B) Quantification of VC by micro-CT (*n* = 8). (C and D) Lipid deposition in en face aortas determined by Oil Red O staining (*n* = 3). (E) Picrosirius Red staining (*n* = 5) and (F) Verhoeff–van Gieson staining (*n* = 5) for assessment of the (G) collagen content and (H) intima–media ratio of the aorta. (I) Fasting blood glucose levels (*n* = 9–10). (J) Body weight gain and final body weight (*n* = 9–10). One-way ANOVA with uncorrected Fisher’s LSD test­(Red Oil O, intima–media ratio, collagen fiber) and two-way ANOVA with Tukey’s multiple comparisons test (Δbody weight) for the data that followed the normal distribution. Brown–Forsythe and Welch ANOVA (blood glucose) was performed for the data that occurred as heterogeneity of variance. The Kruskal–Wallis test with uncorrected Dunn’s test was performed for data that did not follow the normal distribution (AoCS, final body weight). All data are represented as the mean ± SEM. *p < 0.05, **p < 0.01, ***p < 0.001 vs db/db; #*p* < 0.05, ##*p* < 0.01, ###*p* < 0.001 vs WT; *p* > 0.05 n.s.

### 3.4 PA Reduces Macrophage Infiltration and Inflammation in the PVAT of db/db Mice

An increased DVC predicts the atherosclerotic plaque burden. Local PVAT thickness and macrophage infiltration are correlated with the atherosclerotic plaque size and composition in atherosclerosis patients.^39,40^ To elucidate the molecular mechanism of PA in PVAT-associated inflammatory processes in db/db mice, we assessed expression of CD11b and F4/80, which are markers of macrophage infiltration. In the PVAT of db/db mice, we observed an increase in the adipocyte size and substantial macrophage infiltration. Compared to those in WT mice, the number of immunopositive areas and the protein expression of F4/80 and CD11b were obviously greater in the PVAT of db/db mice. PA treatment, particularly at concentrations of 30 and 15 mg/kg, led to a noticeable reduction in CD11b and F4/80 expression, suggesting that PA restrains macrophage infiltration in the PVAT of db/db mice. In addition, we examined the protein expression of the C–C motif chemokine receptor (CCR2), which was elevated in the PVAT of db/db mice, suggesting M1 chemotaxis. PA treatment significantly suppressed CCR2 expression and downregulated tumor necrosis factor-α (TNF-α) mRNA levels and cyclooxygenase-2 (COX-2) protein expression in the PVAT of db/db mice, confirming the potential of PA to selectively target macrophages to reduce inflammatory responses in the PVAT (Figure 4A–E).

**Figure 4:**
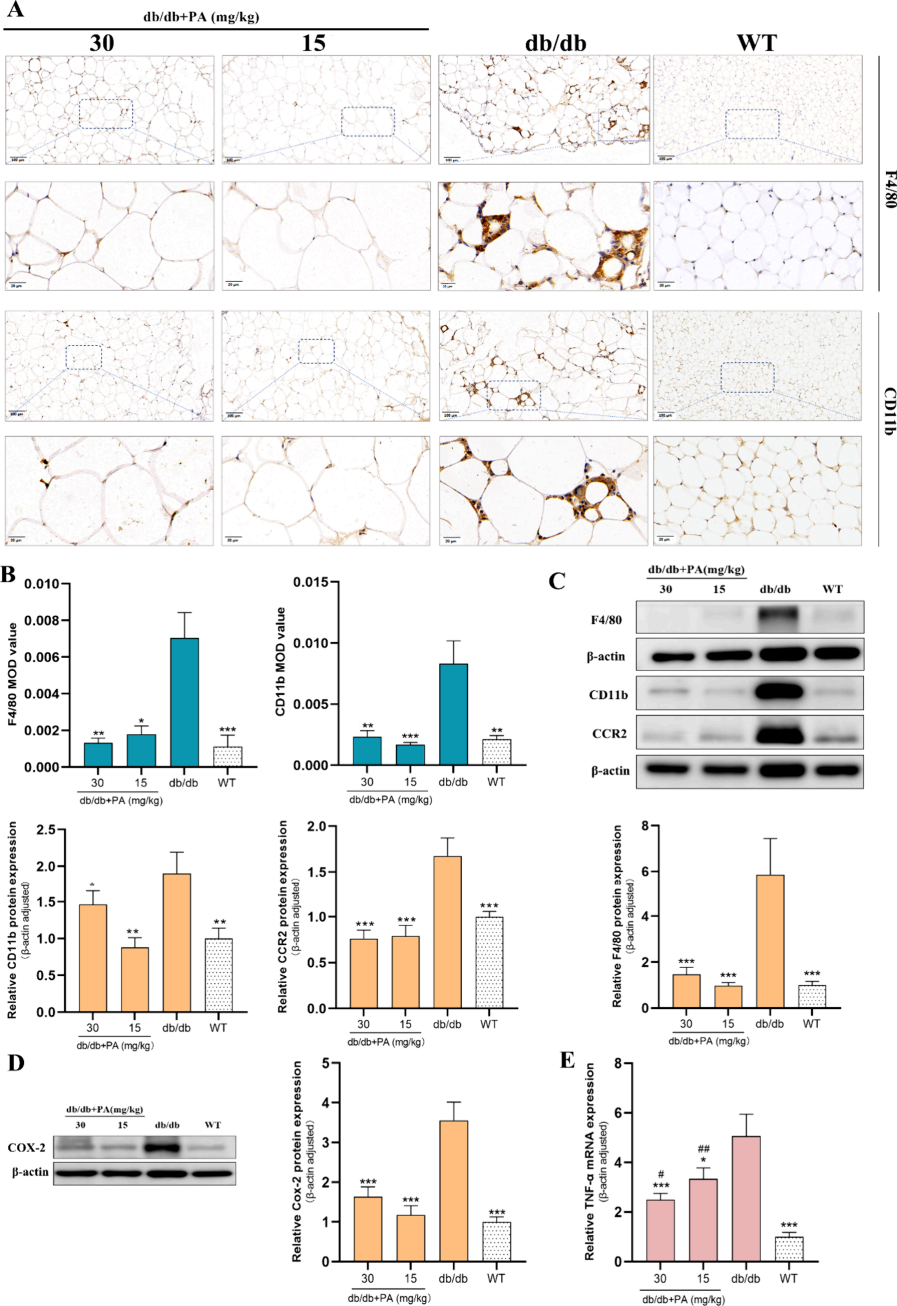
Effect of PA on macrophage infiltration and inflammatory actions in PVAT. (A and B) IHC staining of F4/80 and CD11b. Protein expression of (C) F4/80, CD11b, and CCR2 and (D) COX-2. (E) Evaluation of the mRNA expression of TNF-α. One-way ANOVA with uncorrected Fisher’s LSD test (CCR2, COX-2, and CD11b protein) was performed for the data that followed the normal distribution. Kruskal–Wallis test with uncorrected Dunn’s test was performed for the data that did not follow the normal distribution (IHC of F4/80 and CD11b, F4/80 protein, TNF-α mRNA). All data are represented by statistical significance of mean ± SEM (*n* = 6 for all groups). *p < 0.05, **p < 0.01, ***p < 0.001, #*p* < 0.05, ##*p* < 0.01, ###*p* < 0.001 vs WT, and *p* > 0.05 n.s.

### 3.5 PA Activates Sirt6 in the PVAT of db/db Mice

Sirt6 is an attractive regulatory mechanism for metabolic problems associated with obesity. The adipocyte-specific gene Sirt6 has the potential to modulate IL-4 production and promote M2 polarization of macrophages, which may contribute to the reduction of inflammatory reactions in adipose tissue.^41,42^ To investigate the physiological role of Sirt6 in PVAT-macrophage infiltration in db/db mice, we assessed Sirt6 expression at both the transcript and protein levels. Our results indicated significantly lower Sirt6 mRNA (*p* < 0.001) and protein (*p* < 0.01) expression in the PVAT of db/db mice than in that of WT mice (Figure 5). PA treatment (30 mg/kg) significantly upregulated Sirt6 mRNA expression (*p* < 0.001), nearly restoring it to WT levels (Figure 5A). Similarly, PA treatment (30 and 15 mg/kg) led to a substantial increase in Sirt6 protein expression (*p* < 0.001), corresponding to reduced macrophage infiltration in the PVAT of db/db mice. These findings indicate that PA might exert a stimulatory influence on Sirt6, potentially alleviating proinflammatory responses in PVAT (Figure 5B).

**Figure 5:**
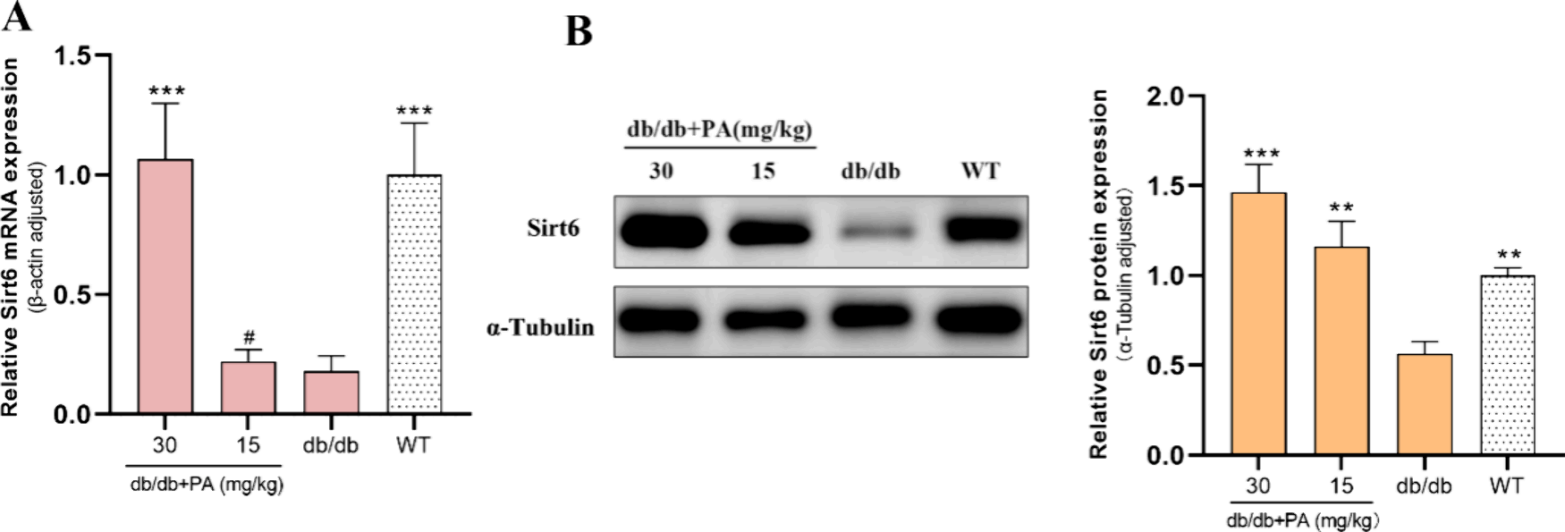
Activation of Sirt6 via PA intervention in the PVAT of db/db mice. (A) Quantification of Sirt6 mRNA expression. (B) Sirt6 protein expression. All data are represented as the mean ± SEM (*n* = 6). The Kruskal–Wallis test with Dunn’s multiple comparisons test was performed for the data that did not follow the normal distribution (Sirt6 mRNA and protein). All data are represented by statistical significance of mean ± SEM (*n* = 6 for all groups). *p < 0.05, **p < 0.01, ***p < 0.001, #*p* < 0.05, ##*p* < 0.01, ###*p* < 0.001 vs WT, and *p* > 0.05 n.s.

## 4 Discussion

Obesity-related diabetes is characterized by the presence of insulin resistance, and dysregulated fatty acid metabolism in an insulin-resistant state elevates the risk of adverse cardiovascular events. Importantly, abnormal adipose expansion in individuals with obesity significantly disrupts the CYP epoxygenase pathway, leading to substantial inhibition of EET within the adipose tissue. Augmenting the effects of EETs has the potential to exert powerful influences on metabolic cardiovascular disease associated with obesity.^43,44^ It is worth noting that, among the EETs, 14,15-EET is highly unstable and rapidly degrades into 14,15-DHET, which exhibits low activity in the presence of sEH. While previous studies have shown the expression of sEH in the epididymal fat pad and liver,^45^ there are no previous reports describing PVAT-derived sEH in db/db mice. In this study, we demonstrated that (a) diabetic obesity was accompanied by the suppression of serum 14,15-EET levels and the marked upregulation of sEH in PVAT and that (b) PA had a protective effect against DVC via a mechanism involving the activation of Sirt6 to inhibit the activity of PVAT-derived sEH. These findings provide unusual insight into the implications of abnormal fatty acid metabolism and sEH in obesity-associated diabetes and the medical value of PA intervention for DVC prevention.

Clinical studies have shown a clear association between diabetes and an increased incidence of VC.^2,28,46^ Additionally, PVAT is related to the calcification of vascular beds. However, the precise underlying mechanism remains elusive. Given the heterogeneity of adipose tissue, it is essential to determine whether the elevated sEH derived from PVAT influences calcification locally or systemically in db/db mice. We observed the presence of sEH both in differentiated 3T3-L1 adipocytes and in PVAT of db/db mice, and the notable increase in sEH in PVAT was accompanied by a reduction in the 14,15-EET levels in db/db mice, which correlated with significant body weight gain compared with that in WT mice. Furthermore, our findings are in accordance with clinical studies. CT analysis revealed irregular calcified deposits primarily in the aortic arch with minimal deposition in the ventral aorta or descending aorta. Compared to that in WT mice, the AoCS in db/db mice were obviously greater. Pathological staining further demonstrated calcification of the intima–medial thickness, accompanied by lipid accumulation, collagen hyperplasia, and elastic fiber rupture in the aortas of db/db mice. In contrast, the aortas of the WT mice did not exhibit vascular structural damage. PVAT typically experiences volume expansion and malfunction in vascular diseases, leading to alterations in the cellular composition. These data suggested that diabetic obesity led to a substantial upregulation of PVAT-derived sEH, which might contribute to vascular diseases in db/db mice, providing new insight into the role of PVAT-derived sEH in VC.

Numerous studies have established that immune cell infiltration is a primary driver of PVAT dysfunction, initiating subsequent inflammation, oxidative stress, reduced production of vasoprotective factors derived from adipocytes with relaxing properties, increased paracrine factor production, and hypoxic processes that promote vascular dysfunction.^47,48^ These factors contribute to atherosclerosis and VC. Notably, sEH upregulation is known to promote inflammatory responses and oxidative stress.^49^ Given that VC is associated with lipid-laden macrophages and intimal hyperplasia,^46^ further investigations are warranted to explore whether PVAT-derived sEH is linked to the infiltration of inflammatory macrophages and cytokine production in the pathological expansion of PVAT in db/db mice. Our findings highlight the significant role of inflammatory PVAT-macrophage infiltration, coupled with sEH upregulation, in contributing to DVC and related vascular pathologies.

Inhibiting sEH to enhance the bioavailability of 14,15-EET is a promising strategy for modulating vascular tone, local inflammation, oxidative stress, insulin sensitivity, and lipid metabolism in obese adipose tissue.^49–51^ Although studies have examined the effects of ginseng extract on CYP1A1 induction mediated by the aryl hydrocarbon receptor,^52^ further investigations of the biological activity and mechanism of PA are needed. Although, as a bioactive ingredient of ginseng, PA is recognized for its antiinflammatory and antioxidant activities, research specifically describing the effects of PA intervention through the sEH-mediated mechanism on AC in db/db mice is currently lacking. In our previous study, we observed only a minor downregulation of sEH in the livers of db/db mice following PA treatment at 30 mg/kg, suggesting that the liver may not be the primary site of PA action. Subsequent experiments demonstrated that PA effectively inhibited sEH activity in differentiated 3T3-L1 adipocytes. In vivo PA treatment reduced sEH expression in PVAT and elevated serum 14,15-EET levels in db/db mice. Furthermore, PA treatment led to a decrease in the AoCS and lipid deposition in the intima layer, while the aortic thickness remained relatively unchanged compared to that of the WT mice. Although the adipocyte size did not significantly decrease, PA markedly reduced the levels of M1 chemotactic macrophages and inflammatory factors (TNF-α and COX-2) in the PVAT of db/db mice. According to these observations, the attenuation of DVC in db/db mice is associated with a reduction in inflammatory macrophages via the inhibition of sEH in PVAT, indicating the potential of sEH inhibition and PA to modulate vascular health.

However, further exploration is needed to fully understand how PA exerts these effects via PVAT-mediated macrophages. As a member of the Sirtuin family, Sirt6 is a nuclear NAD^+^-dependent deacetylase of histones that plays a multifaceted role in regulating various physiological and pathological processes.^53,54^ Studies have shown that Sirt6 deletion results in severe metabolic disorders, including increased insulin resistance and elevated serum triglyceride levels.^55^ Sirt6 has also been implicated in modulating adipose tissue macrophage polarization, transitioning M2-to-M1 macrophages, and exacerbating VC in chronic kidney disease.^42,56^ *Panax ginseng* has been shown to influence proteins of the Sirtuin family,^57,58^ but currently there are limited data on the effect of PA on Sirt6 expression. In vitro experiments demonstrated that PA (6.25 μM) intervention in differentiated 3T3-L1 adipocytes increased Sirt6 mRNA levels (Figure S4). Consequently, we hypothesize that Sirt6 regulation in PVAT may play a pivotal role in the ability of PA to inhibit PVAT-derived sEH and reduce inflammatory macrophage infiltration, ultimately improving DVC. Our results further support that Sirt6 expression at both the protein and transcriptional levels is markedly reduced in the PVAT of db/db mice but significantly upregulated following 30 mg/kg PA treatment. Additionally, PA treatment increased the protein expression of PVAT-derived Sirt6, accompanied by a substantial decrease in fasting blood glucose levels and inflammatory macrophage infiltration. The data presented above indicate that Sirt6 is a potential regulator of PA-mediated effects. Although the exact mechanisms underlying Sirt6 activation in PVAT and its direct or indirect regulation of sEH expression require further investigation, our findings suggest that, in the context of DVC, PA concurrently upregulates PVAT-derived Sirt6 and serum 14,15-EET. This modulation of sEH by PA may serve as a critical mechanism that mediates the balance of lipid metabolism and vascular homeostasis while alleviating macrophage-derived inflammation within a high-glucose environment via Sirt6 activation.

Overall, our study provided evidence that PA exerts a protective effect against DVC by activating Sirt6 to inhibit the activity of PVAT-derived sEH. PA treatment effectively regulates lipid metabolism, maintaining vascular homeostasis and function. These findings enhance our understanding of VC in diabetes and, for the first time, highlight the potential health benefits of PA in DVC management. This research provides novel insights into the mechanisms underlying DVC and the pharmacological implications of PA for cardiovascular health. However, given the complexity of adipose tissue-related calcification and the diverse complications in diabetic patients, further studies are needed to explore the intricate pathways involved and fully assess the therapeutic potential of PA in mitigating DVC and related vascular disorders.

## Supplementary Material



## Notes

Additional data and materials that complement the main manuscript, including structural characterization of PA via ^1^H and ^13^C NMR spectra (Table S1 and Figure S1), 14,15-EET quantitative data (Table S2), primers used in this study (Table S3), effect of PA treatment on 3T3-L1 adipocytes differentiation, lipid accumulation, sEH protein expression, and Sirt6 mRNA levels (Figures S2–S4), and mass of organs and the ratio of organs/body weight of mice (Figure S5) (PDF)

## Abbreviations

ADDs: antidiabetic drugs
AoCS: aortic calcification score
BSA: bovine serum albumin
CCR2: C–C motif chemokine receptor 2
COX-2: cyclooxygenase-2
CYP: cytochrome P450
DHETs: dihydroxyeicosatrienoic acids
DM: diabetes mellitus
DVC: diabetes-induced vascular calcification
EETs: epoxyeicosatrienoic acids
15-EET: (±)­14­(15)-epoxy-5*Z*,8*Z*,11*Z*-eicosatrienoic acid
IBMX: 3-isobutyl-1-methylxanthine
MTBE: methyl *tert*-butyl ether
MTT: thiazolyl blue tetrazolium bromide
PA: panaxynol
PSG: penicillin–streptomycin–glutamine
PVAT: perivascular adipose tissue
qRCR: quantitative real-time PCR
sEH: soluble epoxide hydrolase
TNF-α: tumor necrosis factor-α
VC: vascular calcifying
WT: wild type
