# Supplementary material for: Long-Term In Vivo Administration of Panaxynol Alleviates Diabetes-Induced Vascular Calcification by Modulating Sirt6-Mediated sEH Function in Perivascular Adipose Tissue
Source: J Agric Food Chem. 2025 Jul 7;73(29):18268–79. doi: 10.1021/acs.jafc.5c01982 (PMC12291596; doi:10.1021/acs.jafc.5c01982)
Supplement: Supplementary file 1 [file jf5c01982_si_001.pdf]

Long-term in vivo administration of panaxynol alleviates diabetes-induced vascular calcification by modulating Sirt6-mediated sEH function in perivascular adipose tissue

Shanshan Song [a, d †], Xina Yu [a, e †], Changming Xie [b], Zhanhua Li [a], Ying Zhang [a, c], Yan Liu [c], Zhongjue Qiu [a], Tiantian Wang [a], Hongna Su [a], Hui Huang [b], Pei Luo [a *]

[a] *State Key Laboratory for Quality Research in Chinese Medicines, Guangdong-Hong Kong-Macao Universities Joint Laboratory for Internationalization of TCM, Macau University of Science and Technology, Macau 999078, China*

[b] *Department of Cardiology, The Eighth Affiliated Hospital, Joint Laboratory of Guangdong-Hong Kong-Macao Universities for Nutritional Metabolism and Precise Prevention and Control of Major Chronic Diseases, Sun Yat-sen University, Shenzhen 518033, China.*

[c] *Department of Anesthesiology, The Affiliated TCM Hospital of Southwest Medical University, Luzhou 646000, China*

[d] *Guangxi Liuyao Group Co., LTD, Liuzhou 545001, China*

[e] *School of Biological and Chemical Engineering, NingboTech University, Ningbo 315100, China*

† The authors Shanshan Song and Xina Yu contributed equally to this article.

*Corresponding author:

*Dr. Pei Luo, Tel: +853-88972402; E-mail address: pluo@must.edu.mo; Postal addresses: I01-112, State Key Laboratory for Quality Research in Chinese Medicines, Guangdong-HongKong-Macao Universities Joint Laboratory for Internationalization of TCM, Macau University of Science and Technology, Macau 999078, China. ORCID: 0000-0003-3095-9223.

Supplementary data A

Table captions

Table. S1 ^{1}H and ^{13}C NMR data of panaxynol (δ in ppm, *J* in Hz).

Table. S2 Quantification of 14,15-EET levels in serum (n=5).

Table. S3 The primers for qPCR analysis.

Table. S1

Position	δ_C	δ_H
1	117.06	a, 5.24, d, 1H (10.2)<br>b, 5.47, d, 1H (16.8)
2	136.14	5.94, ddd, 1H (15.6, 10.2, 5.4)
3	63.55	4.92, d, 1H (5.4)
4	74.22	-
5	71.32	-
6	64.01	-
7	80.31	-
8	17.70	3.03, d, 2H (7.2)
9	121.90	5.38, m, 1H
10	133.14	5.51, m, 1H
11	27.21	2.03, dt, 2H (7.2, 7.2)
12	29.24	
13	29.19	
14	29.16	1.27-1.35, m, 10H
15	31.83	
16	22.66	
17	14.11	0.88, t, 3H (6.6)

^{1}H (600 MHz) and ^{13}C (150 MHz) NMR in $CDCl_3$

Table. S2

Group	Peak area	Concentration (ng/ml)	Mean of concentration	Std. Error of Mean
	180	1.086		
	462	2.625		
30 mg/kg	707	3.962	3.439	0.710
	894	4.982		
	813	4.540		
	193	1.157		
	183	1.102		
15 mg/kg	298	1.730	2.677	0.839
	904	5.037		
	780	4.360		
	140	0.867		
	367	2.106		
db/db	131	0.818	1.657	0.474
	588	3.312		
	197	1.179		
	1462	8.082		
	497	2.816		
WT	1837	10.129	5.445	1.530
	554	3.127		
	544	3.072		

Table. S3

Gene	Sequence 5´-3´
**Sirt6**	F: TGTGTTGTCCAGAGGTGAGG
	R: TGCAAGCCTCTACTGATCCC
**sEH**	F: CCATAAGTCAAATATTCAGCCAAG
	R: TATCAGGAAGTCAAAGTGTTGG
**TNF-α**	F: TCTTCTCATTCCTGCTTGTGG
	R: GAGGCCATTTGGGAACTTCT
**β-actin**	F: GATTACTGCTCTGGCTCCTAGCA
	R: GCCACCGATCCACACAGAGT

Supplementary data B

Figure captions

Figure. S1 Identification of PA. **A)** The structure of PA. **B)** ^{1}H- and ^{13}C-NMR spectra of PA .

Figure. S2 Effect of PA treatment on cell differentiation and lipid accumulation. **A-B)** Assessment of cell lipid content by Oil red O staining. **C)** Determination of cell triglyceride content. One-way ANOVA with uncorrected Fisher's LSD test (triglyceride) is performed for the data that followed the normal distribution. Kruskal-Wallis test with uncorrected Dunn's test (lipid) is performed for data that did not follow the normal distribution. All data are represented as the mean ± SEM (n=3). *$p < 0.05$, **$p < 0.01$, ***$p < 0.001$ vs. Ctrl, $p > 0.05$ n.s.

Figure. S3 Effect of PA treatment on sEH protein expression in differentiated 3T3-L1 adipocytes. All data are represented as the mean ± SEM (n=3). Kruskal-Wallis test with uncorrected Dunn's test is performed for data that did not follow the normal distribution. *$p < 0.05$, **$p < 0.01$, ***$p < 0.001$ vs. Ctrl, $p > 0.05$ n.s.

Figure. S4 Effect of PA (1, 2 and 6.25 μM) treatment on Sirt6 mRNA expression in differentiated 3T3-L1 adipocytes. All data are represented as the mean ± SEM (n=3). One-way ANOVA with uncorrected Fisher's LSD test is performed for the data that followed the normal distribution. *$p < 0.05$, **$p < 0.01$, ***$p < 0.001$ vs. Ctrl, $p > 0.05$ n.s.

Figure. S5 Determination of **A)** heart **B)** spleen **C)** liver and **D)** kidney mass and the ratios of body weight. All data are represented as the mean ± SEM (n=8~ 10). One-way ANOVA with uncorrected Fisher's LSD test (heart, liver and kidney) is performed for the data that followed the normal distribution. Kruskal-Wallis test with uncorrected Dunn's test (spleen) is performed for data that did not follow the normal distribution. *$P < 0.05$, **$P < 0.01$, ***$P < 0.001$ vs. db/db group; #$P < 0.05$, ##$P < 0.01$, ###$P < 0.001$ vs. WT, $P > 0.05$ n.s.

Triplicates of original western blots

Figure. S1-A

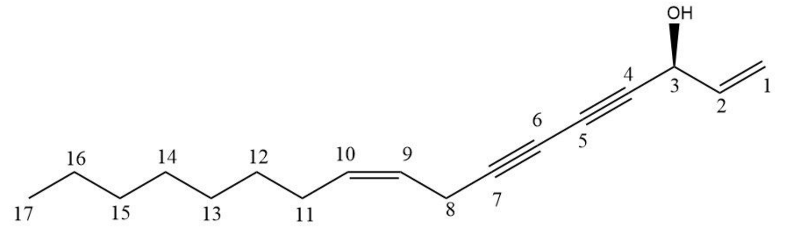

Figure. S1-B

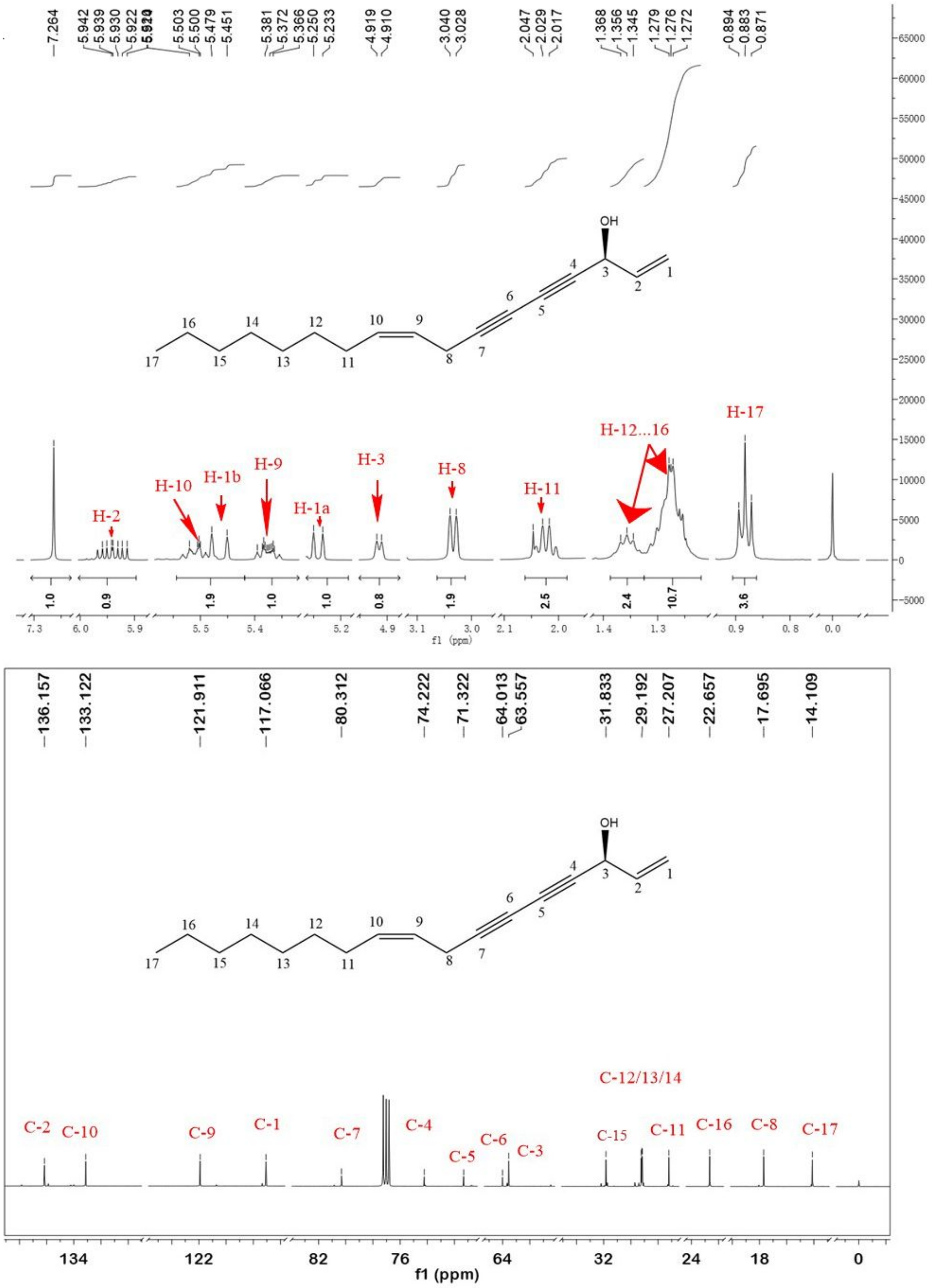

7.264
5.942
5.939
5.930
5.922
5.503
5.500
5.479
5.451
5.381
5.372
5.366
5.250
5.233
4.919
4.910
3.040
3.028
2.047
2.029
2.017
1.368
1.356
1.345
1.279
1.276
1.272
0.894
0.883
0.871
OH
H-2
H-10
H-1b
H-9
H-1a
H-3
H-8
H-11
H-12...16
H-17
1.0
0.9
1.9
1.0
1.0
0.8
1.9
2.5
2.4
10.7
3.6
f1 (ppm)
136.157
133.122
121.911
117.066
80.312
74.222
71.322
64.013
63.557
31.833
29.192
27.207
22.657
17.695
14.109
C-12/13/14
C-2
C-10
C-9
C-1
C-7
C-4
C-5
C-6
C-3
C-15
C-11
C-16
C-8
C-17
f1 (ppm)

Figure. S2

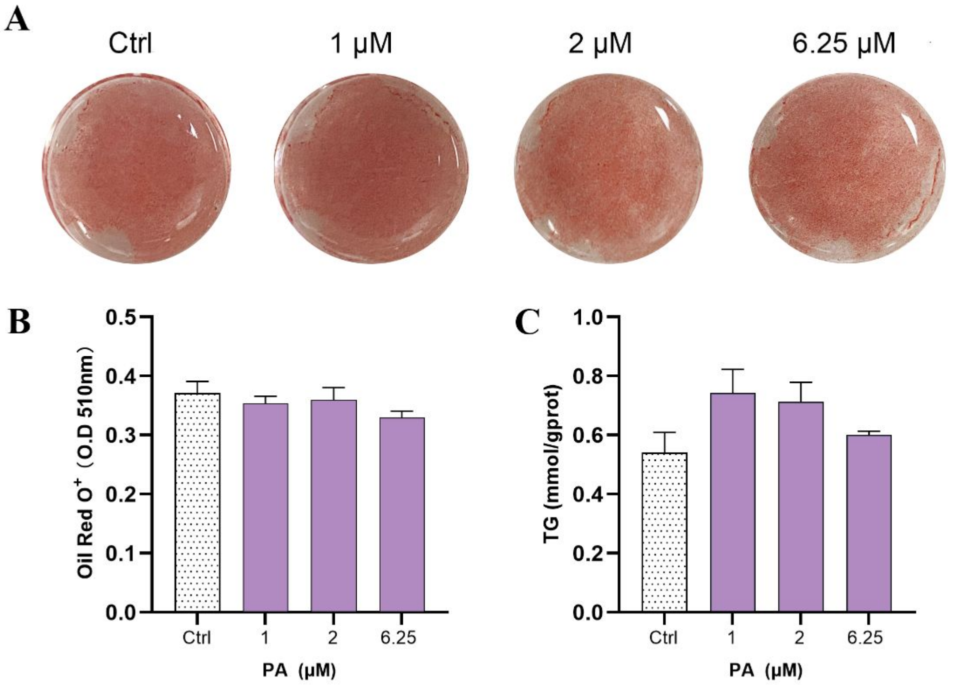
A
Ctrl
1 μM
2 μM
6.25 μM
B
Oil Red O+（O.D 510nm）
0.0
0.1
0.2
0.3
0.4
0.5
Ctrl
1
2
6.25
PA (μM)
C
TG (mmol/gprot)
0.0
0.2
0.4
0.6
0.8
1.0
Ctrl
1
2
6.25
PA (μM)

Figure. S3

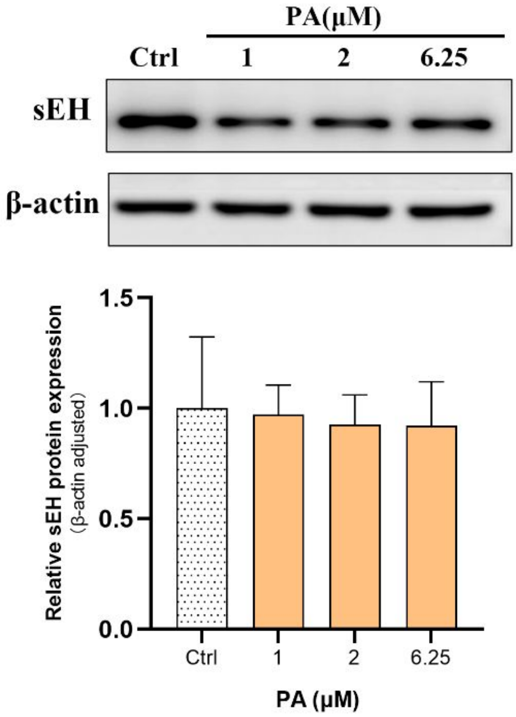
PA(μM)
Ctrl
1
2
6.25
sEH
β-actin
Relative sEH protein expression
(β-actin adjusted)
1.5
1.0
0.5
0.0
Ctrl
1
2
6.25
PA (μM)

Figure. S4

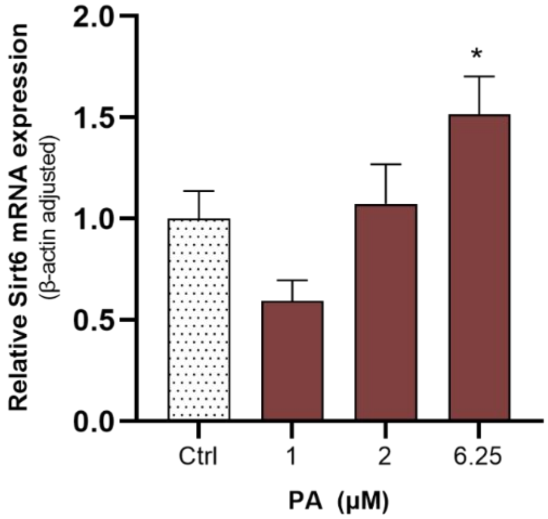
Relative Sirt6 mRNA expression
(β-actin adjusted)
2.0
1.5
1.0
0.5
0.0
*
Ctrl
1
2
6.25
PA (μM)

Figure. S5

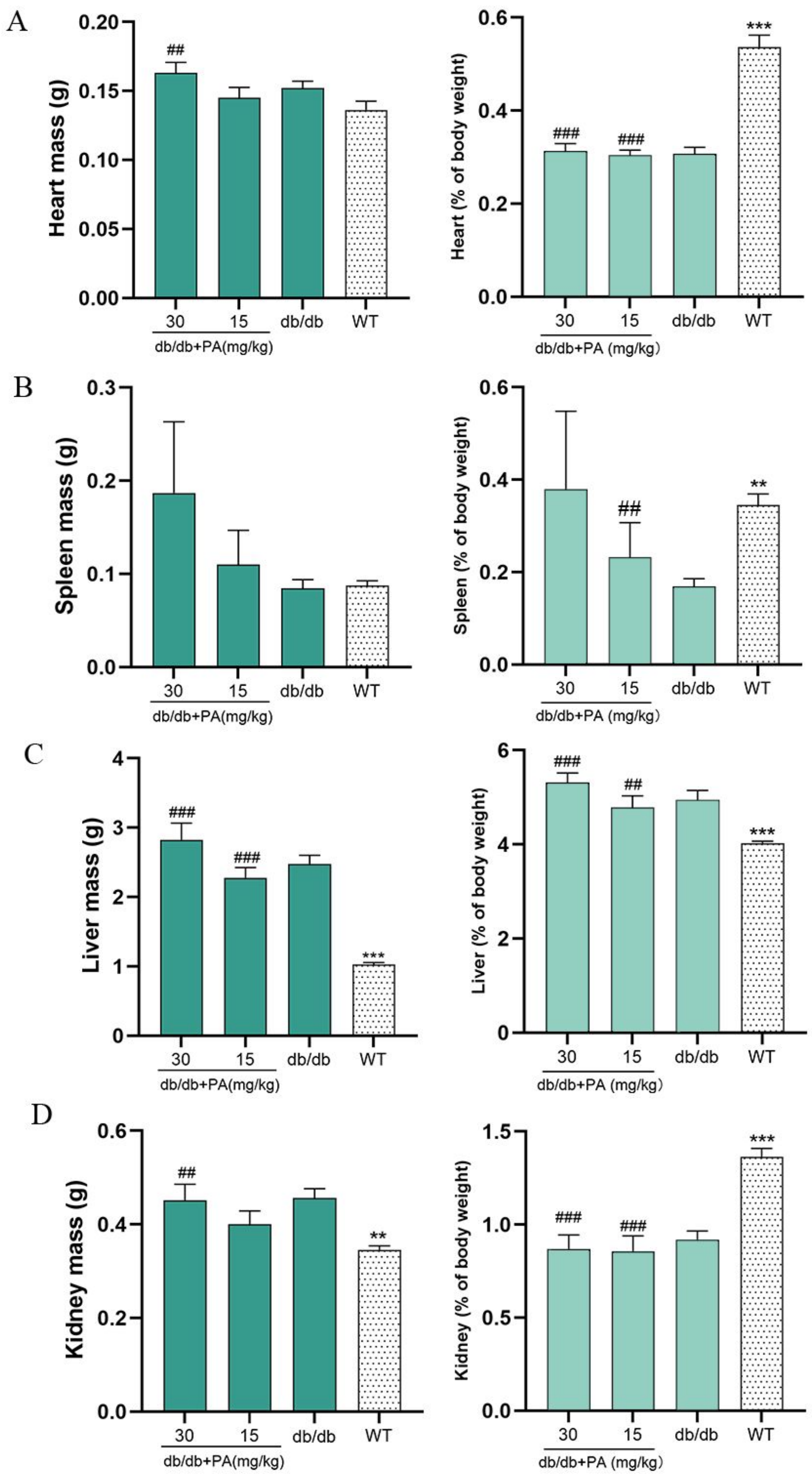
A
Heart mass (g)
Heart (% of body weight)
B
Spleen mass (g)
Spleen (% of body weight)
C
Liver mass (g)
Liver (% of body weight)
D
Kidney mass (g)
Kidney (% of body weight)
30
15
db/db
WT
db/db+PA(mg/kg)
##
###
**
***

Triplicates of original western blots

Figure 2C

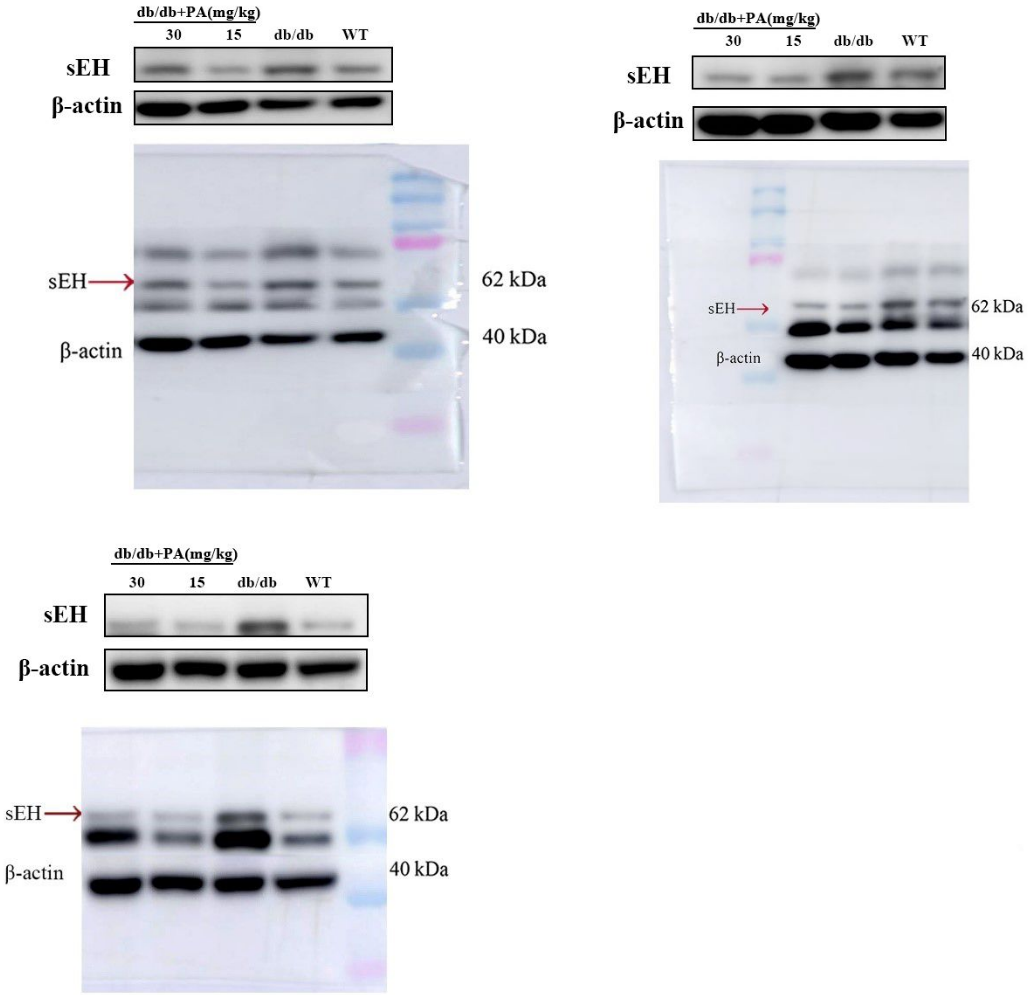

Figure 4C

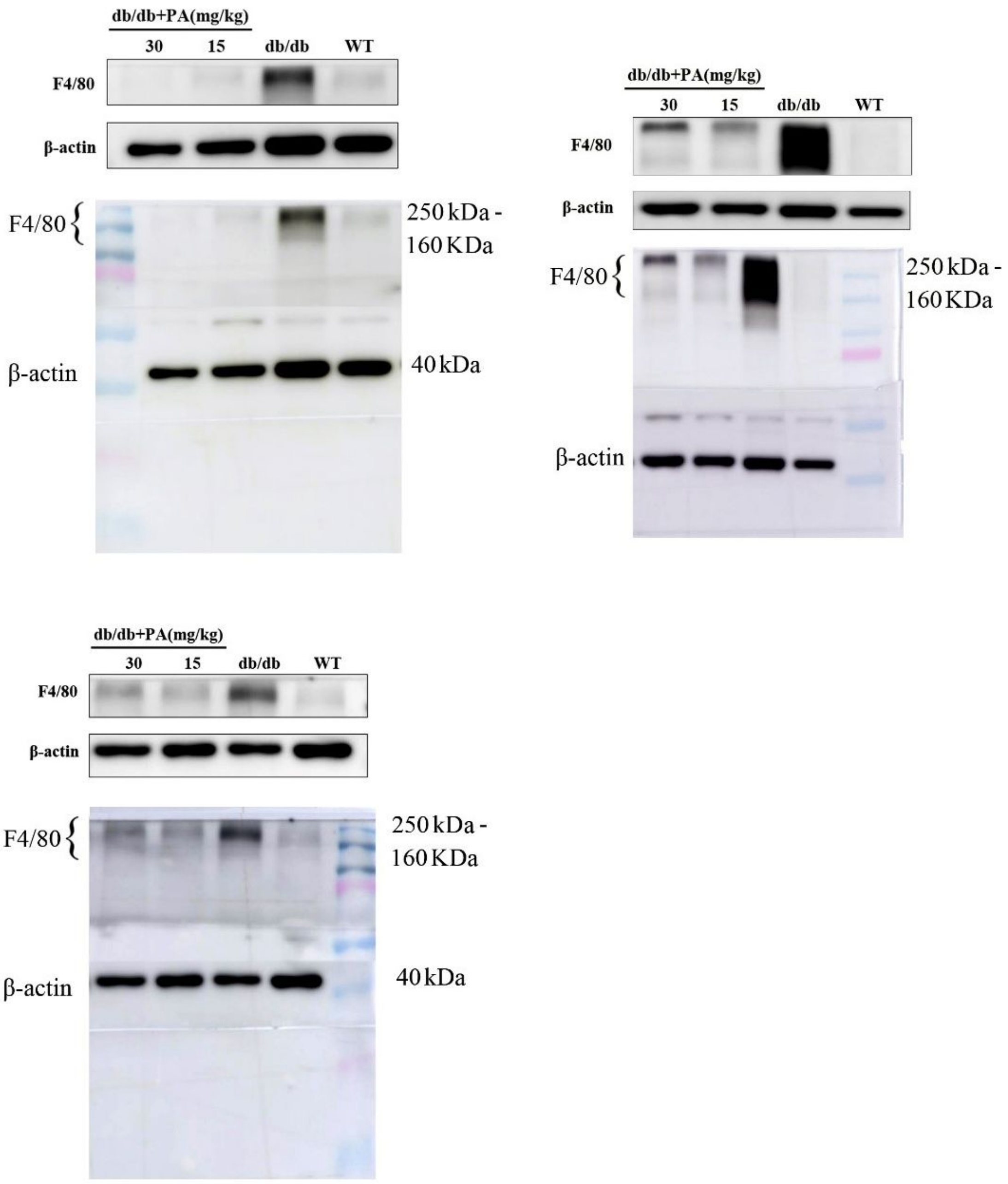
db/db+PA(mg/kg)
30
15
db/db
WT
F4/80
β-actin
F4/80
250 kDa -
160 KDa
β-actin
40 kDa
db/db+PA(mg/kg)
30
15
db/db
WT
F4/80
β-actin
F4/80
250 kDa -
160 KDa
β-actin
db/db+PA(mg/kg)
30
15
db/db
WT
F4/80
β-actin
F4/80
250 kDa -
160 KDa
β-actin
40 kDa

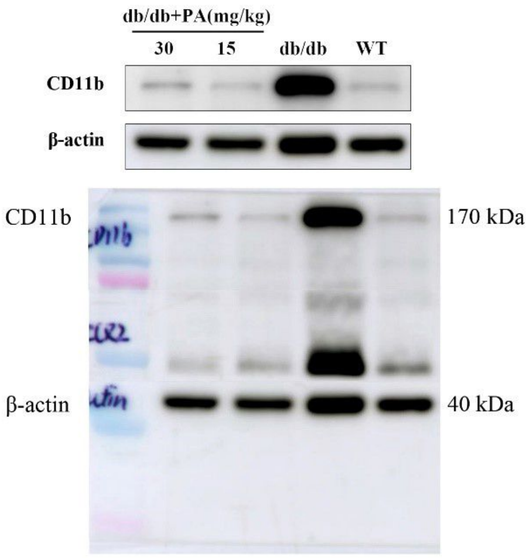
db/db+PA(mg/kg)
30
15
db/db
WT
CD11b
β-actin
CD11b
170 kDa
β-actin
40 kDa

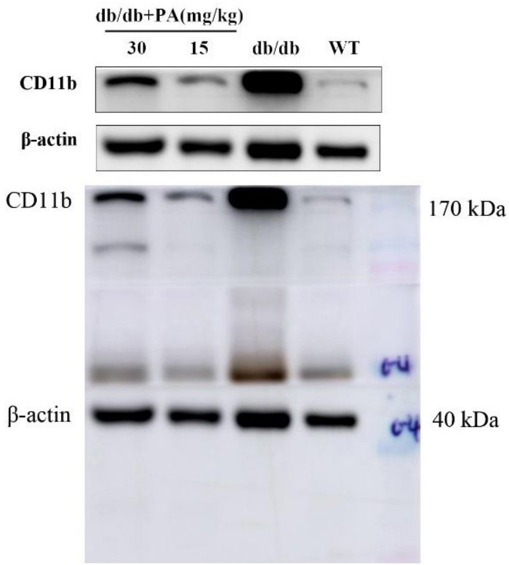
db/db+PA(mg/kg)
30
15
db/db
WT
CD11b
β-actin
CD11b
170 kDa
β-actin
40 kDa

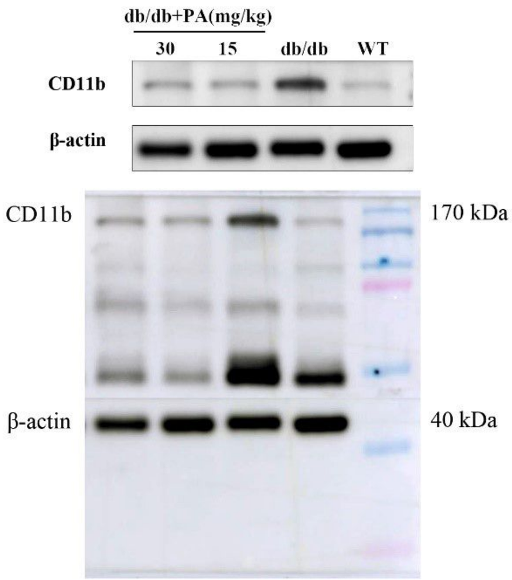
db/db+PA(mg/kg)
30
15
db/db
WT
CD11b
β-actin
CD11b
170 kDa
β-actin
40 kDa

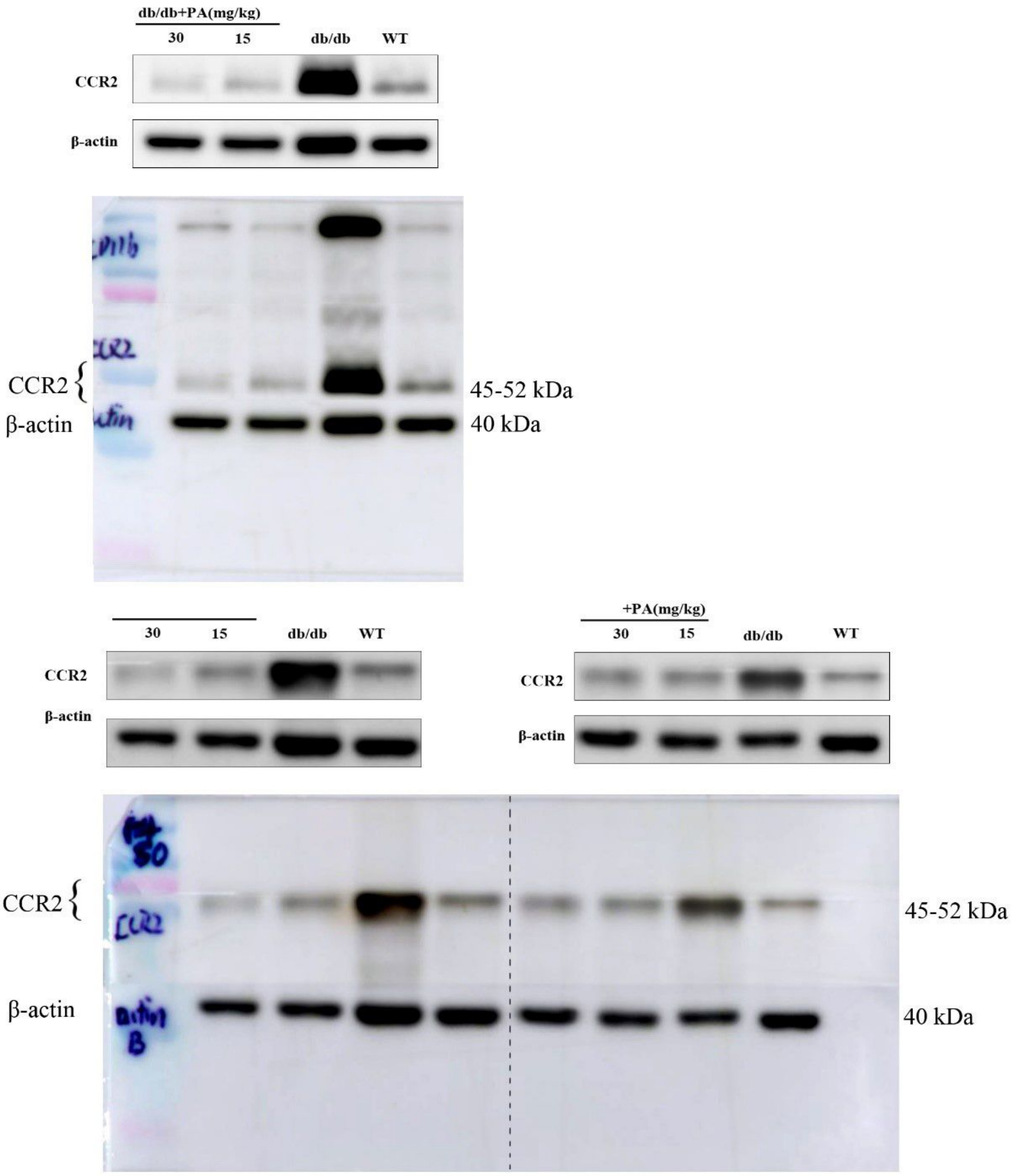
db/db+PA(mg/kg)
30
15
db/db
WT
CCR2
β-actin
CCR2
β-actin
45-52 kDa
40 kDa
30
15
db/db
WT
CCR2
β-actin
+PA(mg/kg)
30
15
db/db
WT
CCR2
β-actin
CCR2
β-actin
45-52 kDa
40 kDa

Figure 4D

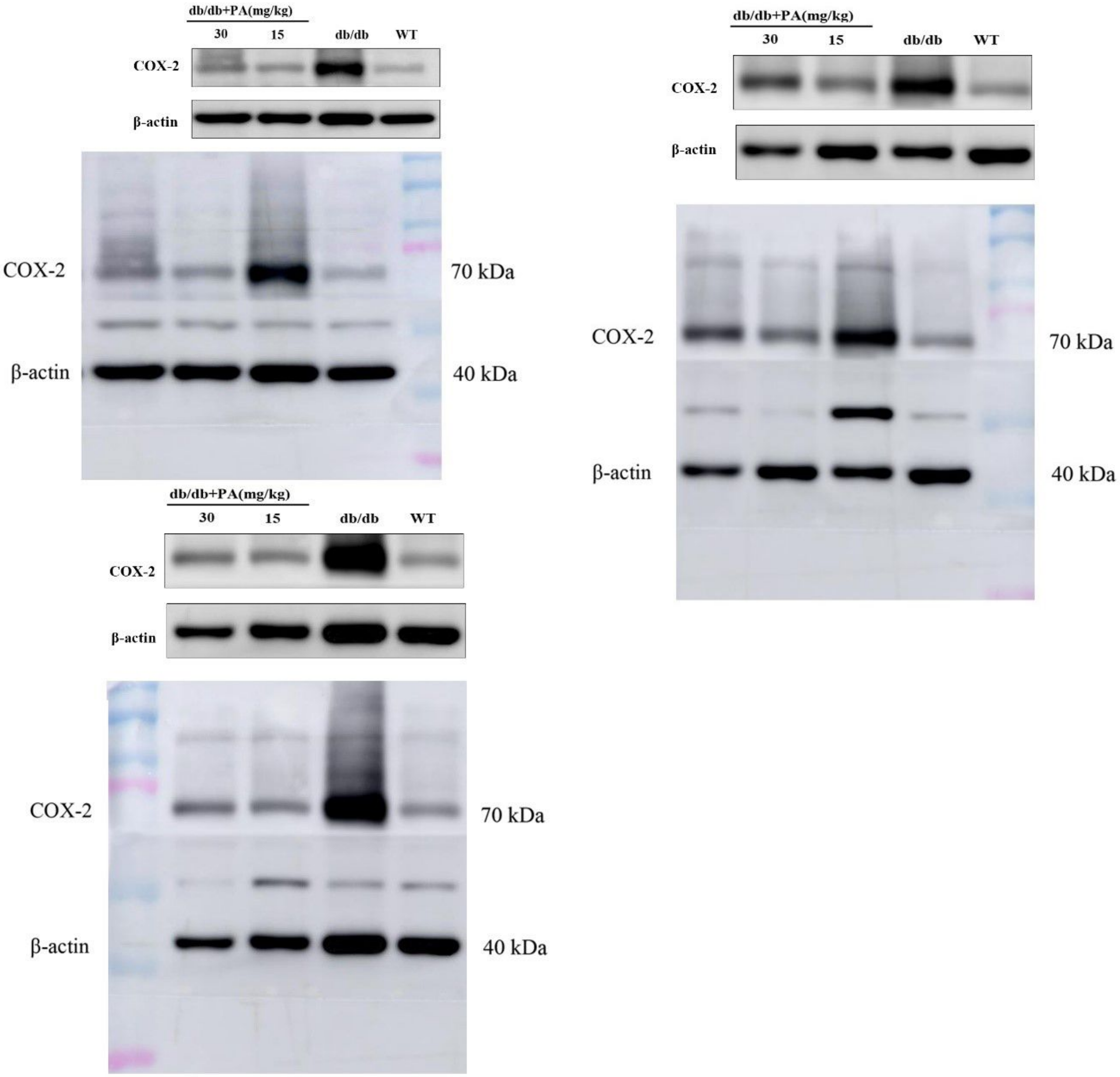

db/db+PA(mg/kg)
30
15
db/db
WT
COX-2
β-actin
COX-2
70 kDa
β-actin
40 kDa
db/db+PA(mg/kg)
30
15
db/db
WT
COX-2
β-actin
COX-2
70 kDa
β-actin
40 kDa
db/db+PA(mg/kg)
30
15
db/db
WT
COX-2
β-actin
COX-2
70 kDa
β-actin
40 kDa

Figure 5B

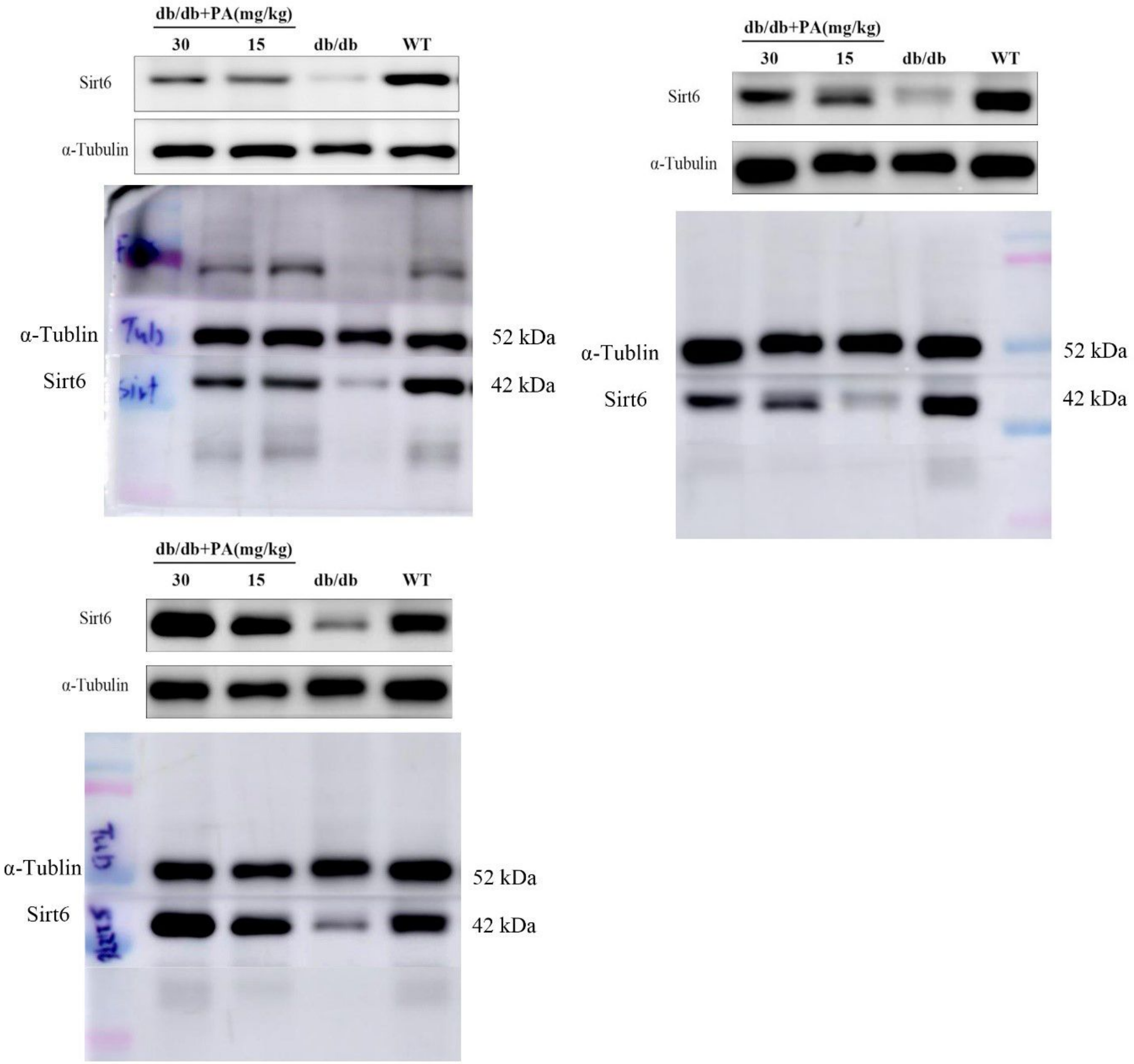
db/db+PA(mg/kg)
30
15
db/db
WT
Sirt6
α-Tubulin
α-Tublin
52 kDa
42 kDa

Figure S3

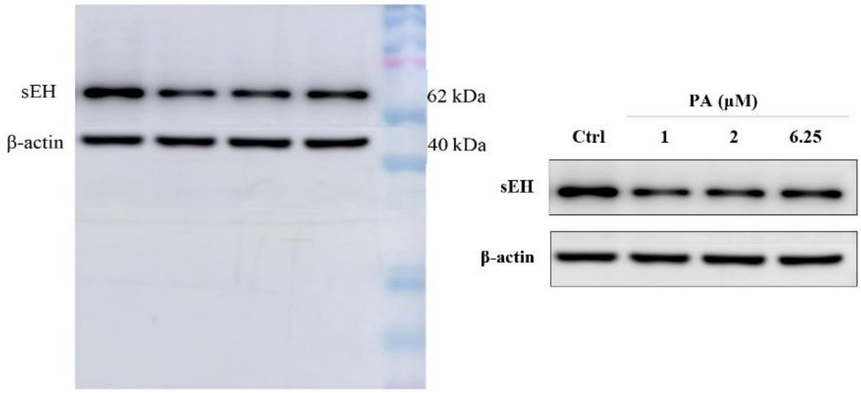
sEH
β-actin
62 kDa
40 kDa
PA (μM)
Ctrl
1
2
6.25
sEH
β-actin

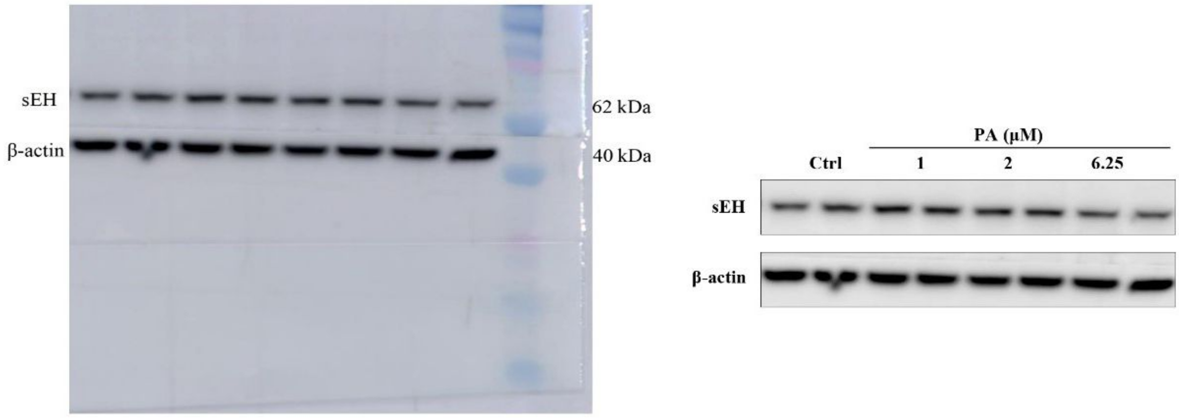
sEH
β-actin
62 kDa
40 kDa
PA (μM)
Ctrl
1
2
6.25
sEH
β-actin
